# Genome-Wide Study of Colocalization between Genomic Stretches: A Method and Applications to the Regulation of Gene Expression

**DOI:** 10.3390/biology11101422

**Published:** 2022-09-29

**Authors:** Yuri V. Kravatsky, Vladimir R. Chechetkin, Nickolai A. Tchurikov, Galina I. Kravatskaya

**Affiliations:** 1 Engelhardt Institute of Molecular Biology, Russian Academy of Sciences, Vavilov Str., 32, 119991 Moscow, Russia; 2 Center for Precision Genome Editing and Genetic Technologies for Biomedicine, Engelhardt Institute of Molecular Biology, Russian Academy of Sciences, 119991 Moscow, Russia

**Keywords:** GWAS, genome tracks, epigenetics, stretches, biostatistics, bioinformatic tool, CpG islands (CGI), transcription start site (TSS), histone mark H2A.Z

## Abstract

**Simple Summary:**

Addressing a large number of genomic problems requires the comparison of genetic and epigenetic features distributed over the genome (genome tracks). The mutual arrangement of these features determines basic molecular mechanisms related to the dynamics of the genome architecture and gene expression. The analysis of data on the genome tracks stored in numerous databases cannot be performed without suitable bioinformatic tools. A package, the Genome Track Colocalization Analyzer, developed by the authors, is intended for the study of colocalization effects between stretch–stretch and stretch–point genome tracks.

**Abstract:**

In this paper, we describe a method for the study of colocalization effects between stretch–stretch and stretch–point genome tracks based on a set of indices varying within the (–1, +1) interval. The indices combine the distances between the centers of neighboring stretches and their lengths. The extreme boundaries of the interval correspond to the complete colocalization of the genome tracks or its complete absence. We also obtained the relevant criteria of statistical significance for such indices using the complete permutation test. The method is robust with respect to strongly inhomogeneous positioning and length distribution of the genome tracks. On the basis of this approach, we created command-line software, the Genome Track Colocalization Analyzer. The software was tested, compared with other available packages, and applied to particular problems related to gene expression. The package, Genome Track Colocalization Analyzer (GTCA), is freely available to the users. GTCA complements our previous software, the Genome Track Analyzer, intended for the search for pairwise correlations between point-like genome tracks (also freely available). The corresponding details are provided in Data Availability Statement at the end of the text.

## 1. Introduction

The development of next-generation sequencing (NGS) technology (for a review and further references, see, e.g., [1]) caused the explosive growth of experimental data for genomes of various organisms, cellular lines, and tissues. These data are collected in numerous databases (ENCODE [2], EPD [3], GENCODE [4], NCBI GEO [5], FANTOM5 project [6], etc.). A detailed study of the architecture and functioning of the genome in the context of different genetic and epigenetic features cannot be performed without special bioinformatic tools (reviewed in [7]). Formally, the study of the coordination between related genetic and epigenetic features can be reduced to statistical analysis of the point-like and stretch-like objects distributed over the genome. Our previous publication [8] was devoted to the first part of this problem (i.e., to the analysis of point-like objects), whereas in this paper, we present a method for the analysis of stretch–stretch and stretch–point colocalization. As stretches are objects with two degrees of freedom (corresponding to the positioning of centers or any other characteristic point and lengths of stretches), it is reasonable, first, to analyze the coordinated positioning of stretch centers with our Genome Track Analyzer (GTA) package [8], and then to extend the analysis further using the methods presented in this paper. To suppress outliers, GTA applies locally normalized distances between neighboring points. The randomness of point distribution for a particular set is initially assessed with entropy-like estimation. The resulting output is based on the statistically tested theoretically derived criterion.

The positioning of stretches over the genome is commonly strongly inhomogeneous, and the distribution of their lengths is also far from the standard statistical distributions. Therefore, in available packages including GenometriCorr [9], GAT [10], regioneR [11], LOLA [12], and GIGGLE [13], partial permutation tests are performed for the assessment of stretch–stretch colocalization. The optimal choice of sampling in a partial permutation test is ambiguous and at present remains a matter of discussion [14,15,16]. The convergence of the statistical thresholds for the partial sampling is usually not checked. Moreover, the proof of convergence by simulations would be time-consuming.

Let us briefly characterize the available packages [9,10,11,17] which are compared below with our tool for the study of colocalization between genomic stretches. The packages [9,10,11] are intended exclusively for the study of overlapping between stretches. The potential correlations in the positioning of non-overlapping stretches are ignored. The same applies to the opposite case when one of the stretches is completely located inside the other. GenometriCorr [9] applies the global Jaccard coefficient for the assessment of overlapping between stretches, whereas GAT [10] and regioneR [11] employ measures in raw non-normalized lengths. All these measures are sensitive to outliers. Because statistical outliers may be biologically meaningful, methods that are robust to outliers are preferable. StereoGene [17] is intended for rapid estimation of genome-wide correlations among pairs of genomic features and/or genome-wide profiles and does not provide information about overlapping. The final results provided by packages [9,10,11,17] are based on the comparison of genomic sets versus randomized ones using the partial permutation test for the assessment of the statistical significance of colocalization effects. The parameters for the partial permutation test and randomization trials were either determined by the developers or may be set by the user.

Our approach is based on an original set of indices characterizing stretch–stretch and stretch–point colocalization and on the complete permutation test, which is free from the above-mentioned ambiguity. The analytical criteria that we derive for the complete permutation test do not need re-sampling simulations and ensure the fast speed of computations. The Genome Track Colocalization Analyzer (GTCA) package, developed on the basis of this theory, was tested, compared with the other available packages, and applied to the study of different mechanisms related to gene expression. Some of the examples in this work illustrate known effects and show that the results of our approach coincide with these known effects, while our genome-wide study of colocalization between tracks for histone H2A.Z (H2AFZ) and transcription start sites (TSSs) in the *Homo sapiens* genome is original and suggests new results. We show that such colocalization is pronounced more strongly for bidirectional promoters in comparison with unidirectional ones and may be considered a distinctive feature of gene expression for bidirectional promoters.

## 2. Theory and Methods

Below, we present the theory and methods for the statistical analysis of stretch–stretch and stretch–point colocalization. Our general approach, theory, and results are original. We begin with the general definitions.

### 2.1. Characterizing Stretch–Stretch and Stretch–Point Characteristics by Sets of Indices

In most genetic problems, the distribution of stretch lengths as well as the distribution of intervals between the centers of neighboring stretches are rather poorly approximated by standard statistical distributions and should be considered as unique. The stretch lengths and intervals between stretches are strongly variable and inhomogeneously distributed over the genome. For this reason, an integral measure for colocalization between stretches should be built from local pairs, while a local measure for colocalization should be constructed from relative characteristics. To solve this problem, we developed sets of local indices characterizing different aspects of colocalization between stretches and robust to the variations of lengths.

In our approach, the colocalization of pairs of stretches of different types was analyzed. In the theory below, we designate the types as A and B.

The indices are defined for the nearest neighbors in the combinations of different neighboring stretches restricted to BAB and ABA. The nearest neighbors were determined by the positions of stretch centers as described previously [8]. All indices vary within the interval (–1, 1); the value –1 corresponds to the strongest colocalization, while the value +1 corresponds to the absence of colocalization. We used the following set of indices:

(i) *The index of overlapping (IO)* characterizes mutual stretch–stretch colocalization and is defined as:
  (1) $$IO_{k}=\frac{|m_{c}(B_{k})-m_{c}(A_{k})|-(a_{k}+b_{k})/2}{|m_{c}(B_{k})-m_{c}(A_{k})|+(a_{k}+b_{k})/2}=\frac{L_{A_{k}B_{k}}-(a_{k}+b_{k})/2}{L_{A_{k}B_{k}}+(a_{k}+b_{k})/2}$$
  where *k* refers to the *k*-th pair of the nearest neighbors, $m_{c}(A_{k})$ and $m_{c}(B_{k})$ denote the sites of stretch centers over the genome, $L_{A_{k}B_{k}}$ is the distance between the centers of neighboring stretches, and *a_k_* and *b_k_* denote the lengths of the stretches.
(ii) *The index of asymmetry (IA)* characterizes the skewness between the lengths of the k-th nearest neighbors:
  (2) $$IA_{k}=\frac{a_{k}-b_{k}}{a_{k}+b_{k}}$$
(iii) *The index of coverage (IC)* characterizes the mutual colocalization between stretches (A) and points (B):
  (3) $$IC_{k}=\frac{|m(B_{k})-m_{c}(A_{k})|-a_{k}/2}{|m(B_{k})-m_{c}(A_{k})|+a_{k}/2}=\frac{L_{A_{k}B_{k}}-a_{k}/2}{L_{A_{k}B_{k}}+a_{k}/2}$$

**Figure 1:**
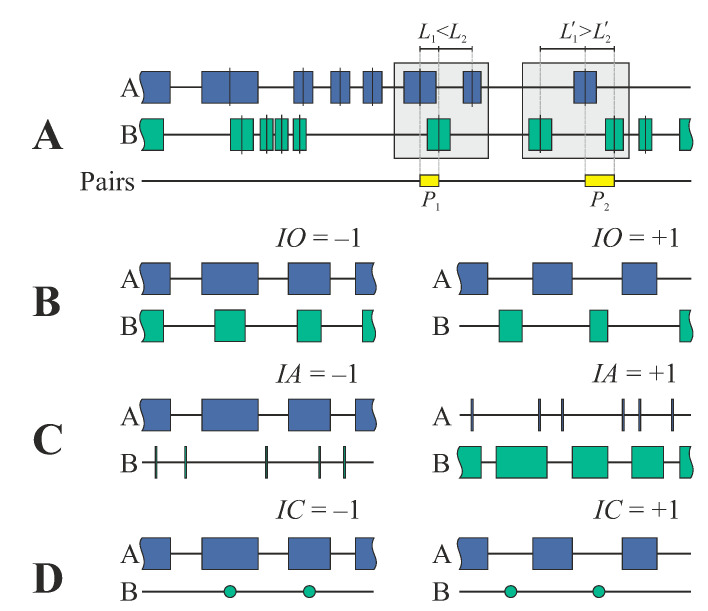
(**A**) Definition of the nearest neighbors between the stretches of two types, A and B. The positions of stretches over the genome and the distances between the stretches are defined by the centers of the stretches. The pairs for analysis of correlations were chosen as the nearest neighbors in the combinations ABA and BAB. (**B**–**D**) Indices for the analysis of mutual stretch characteristics. (**B**) Index of overlapping (Equation (1)) is equal to −1 if the centers of A- and B-stretches coincide with each other (complete colocalization). For remote non-overlapping stretches (absence of colocalization), the index of overlapping tends towards +1. (**C**) Index of asymmetry (Equation (2)) characterizes the difference in the distributions of stretch lengths. If the A-stretches are much shorter in comparison to B-stretches, the index of asymmetry is equal to −1, whereas if the A-stretches are much longer than the B-stretches, it tends towards +1. (**D**) Index of coverage (Equation (3)) is equal to −1 if the centers of A-stretches and positions of B-points coincide with each other (complete colocalization). For remote non-overlapping positioning (absence of colocalization), the index of coverage tends towards +1.

Figure 1 illustrates the relationships between indices and different geometric characteristics of stretches. The mean indices and their squared deviations for *K* pairs of the nearest neighbors are defined as:

(4) $$\bar{I}\;=\frac{1}{K}\sum_{k=1}^{K}I_{k}$$

(5) $$\sigma^{2}(I)\;=\frac{1}{K-1}\sum_{k=1}^{K}{\left(I_{k}-\bar{I}\right)}^{2}$$

### 2.2. Statistical Criteria

A non-parametric assessment of statistical significance for genome-wide associations can be conveniently performed via a permutation test [11,14,15,16]. To exclude the effects related to finite sampling, we used the complete permutation test. For *K* primary pairs of stretches, the complete permutations produce K(K−1)/2 additional different pairs.

The consecutive permutations of the type A and type B stretches determine two classes of permuted indices of overlapping:

(6a) $$IO_{kk^{\prime }}^{\left(a\right)}=\frac{1}{2}\;\left(\frac{L_{A_{k}B_{k}}-(a_{k^{\prime }}+b_{k})/2}{L_{A_{k}B_{k}}+(a_{k^{\prime }}+b_{k})/2}+\frac{L_{A_{k^{\prime }}B_{k^{\prime }}}-(a_{k}+b_{k^{\prime }})/2}{L_{A_{k^{\prime }}B_{k^{\prime }}}+(a_{k}+b_{k^{\prime }})/2}\right)\;\;;\;k\neq k^{\prime }$$

(6b) $$IO_{kk^{\prime }}^{\left(b\right)}=\frac{1}{2}\;\left(\frac{L_{A_{k}B_{k}}-(a_{k}+b_{k^{\prime }})/2}{L_{A_{k}B_{k}}+(a_{k}+b_{k^{\prime }})/2}+\frac{L_{A_{k^{\prime }}B_{k^{\prime }}}-(a_{k^{\prime }}+b_{k})/2}{L_{A_{k^{\prime }}B_{k^{\prime }}}+(a_{k^{\prime }}+b_{k})/2}\right)\;;\;k\neq k^{\prime }$$

In particular, the index $IO_{kk^{\prime }}^{\left(a\right)}$ corresponds to the permutation of the type A stretch from the *k’*-th pair into the position occupied by the A-stretch in the *k*-th pair and vice versa, whereas for the index $IO_{kk^{\prime }}^{\left(b\right)}$, the similar permutation was performed for B-stretches. The criteria for stretch–stretch colocalization were primarily based on the indices (6a) and (6b). In addition to these basic indices, we also used the auxiliary indices obtained by the simultaneous permutations of A and B:

(7) $$IO_{kk^{\prime }}^{\left(2\right)}=\frac{1}{2}\;\left(\frac{L_{A_{k}B_{k}}-(a_{k^{\prime }}+b_{k^{\prime }})/2}{L_{A_{k}B_{k}}+(a_{k^{\prime }}+b_{k^{\prime }})/2}+\frac{L_{A_{k^{\prime }}B_{k^{\prime }}}-(a_{k}+b_{k})/2}{L_{A_{k^{\prime }}B_{k^{\prime }}}+(a_{k}+b_{k})/2}\right)\;;\;k\neq k^{\prime }$$

For the indices of asymmetry and coverage, the permuted counterparts were defined, respectively, as:

(8) $$IA_{kk^{\prime }}\;=\frac{1}{2}\;\left(\frac{a_{k}-b_{k^{\prime }}}{a_{k}+b_{k^{\prime }}}+\frac{a_{k^{\prime }}-b_{k}}{a_{k^{\prime }}+b_{k}}\right);\;\;k\neq k^{\prime }$$

(9) $$IC_{kk^{\prime }}=\frac{1}{2}\;\;\left(\frac{L_{A_{k}B_{k}}-a_{k^{\prime }}/2}{L_{A_{k}B_{k}}+a_{k^{\prime }}/2}+\frac{L_{A_{k^{\prime }}B_{k^{\prime }}}-a_{k}/2}{L_{A_{k^{\prime }}B_{k^{\prime }}}+a_{k}/2}\right)\;;\;\;\;k\neq k^{\prime }$$

The corresponding mean values and variances for all permuted indices were calculated as:

(10) $$<I_{kk^{\prime }}>\;\equiv \;<I{>}_{p}\;=\frac{2}{K(K-1)}\sum_{k=1}^{K-1}\sum_{k^{\prime }=k+1}^{K}I_{kk^{\prime }}$$

(11) $$\sigma^{2}(I_{kk^{\prime }})\;=\frac{1}{K(K-1)/2-1}\sum_{k=1}^{K-1}\sum_{k^{\prime }=k+1}^{K}\left(I_{kk^{\prime }}-<I{>}_{p}\right){\;}^{2}$$

In the calculations of the variance for the difference:

(12) $$\Delta I=\frac{1}{K}\sum_{k=1}^{K}I_{k}-\frac{2}{K(K-1)}\sum_{k=1}^{K-1}\sum_{k^{\prime }=k+1}^{K}I_{kk^{\prime }}\equiv \bar{I}\;-\;<I{>}_{p}$$

the covariance terms appear to be crucial because the same stretches participate in many different permutations. After extensive simulations, we found that two generic drawbacks are inherent to the strict calculations of covariance terms. The number of terms in covariance sums grows proportionally to *K*^3^. Therefore, starting with *K* about 10^3^ and higher, the summation of covariance sums with constraints becomes time-consuming. At the opposite limit of relatively small *K* within the range 50–500, the resulting variance loses robustness and becomes sensitive to particular random realizations. Instead, we found that such drawbacks were absent in the following simplified approximation for the variance:

(13) $$\sigma_{eff}^{2}(\Delta I)\approx \sigma^{2}(I)/K+2\sigma^{2}(I_{kk^{\prime }})/K-2Cov$$

where *K* is the total number of stretch pairs; the variances $\sigma^{2}(I)$ and $\sigma^{2}(I_{kk^{\prime }})$ are defined by Equations (5) and (11), respectively; and:

(14) $$Cov=\frac{2}{K^{2}(K-1)}\sum_{k=1}^{K-1}\sum_{k^{\prime }=k+1}^{K}\left[\;\left(I_{k}-\bar{I}\;\right)\;\left(I_{kk^{\prime }}-<I{>}_{p}\right)+\left(I_{k^{\prime }}-\bar{I}\;\right)\;\left(I_{kk^{\prime }}-<I{>}_{p}\right)\;\right]$$

The resulting criterion was formulated in terms of difference (12) normalized to the effective standard deviation determined by Equation (13):

(15) $$\zeta_{I}=\Delta I/\sigma_{eff}(\Delta I)$$

Negative values of $\zeta_{I}$ correspond to stronger stretch–stretch and stretch–point colocalization for the indices of overlapping and coverage related to genome tracks under analysis in comparison with permuted configurations, and vice versa (the sign of $\zeta_{I}$ is defined as in (1) and (3); see also Figure 1).

The permutation test implies significant variations in stretch lengths. Indeed, if all stretch lengths were identical, the permutations of stretches would become indiscernible. The restriction for the variability of stretch lengths is formulated in terms of coefficient of variation (*CV*) for the lengths:

(16) $$CV=\sigma (a)/\bar{a}\;\;>CV_{thr}$$

where $\bar{a}$ is the mean length of the type A stretches and σ(a) is the standard deviation for lengths. The criterion for the type B stretches is similarly formulated. The threshold *CV_thr_* should be about 0.5–1. If the in Equation (16) is violated for the stretches of a particular type, such stretches should be treated as points by the characteristic starts/centers/ends.

### 2.3. Simulations

**Figure 2:**
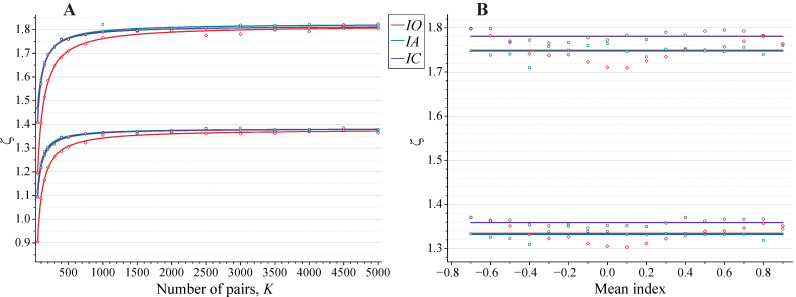
The absolute values of ζ parameters (Equation (15)) corresponding to empirical probabilities Pr = 0.01 (upper curves) and 0.05 (lower curves), as determined by 10^5^ random realizations. Both A- and B-stretches were taken to be random. In this case, the statistics for all permuted overlapping indices (6a), (6b), and (7) were identical within statistical scattering. (**A**) The dependence of ζ parameters on the number of the nearest neighbors *K*. The values of all mean indices were in the vicinity of zero. The solid curves correspond to the best approximation by Equation (17). (**B**) The dependence of ζ parameters on the mean indices $\bar{I}$ (Equation (4)). The number of the nearest neighbors was 580. The dependence on the mean indices of thresholds for ζ parameters was taken as |ζ| = constant. The solid lines correspond to the related best approximations.

Extensive simulations revealed that the statistics for all indices (6)–(9) was approximately universal for random sets. The absolute values of $\left|\zeta_{I}\right|$ for 5% and 1% empirical probability thresholds shown in Figure 2 indicate their monotonic dependence on the number of pairs (Figure 2A) and weak dependence (within statistical scattering) on the mean indices (Figure 2B). The dependence of $\zeta_{I}$ on the number of pairs *K* can, with good accuracy, be approximated by:

(17) $$\zeta_{I}-\zeta_{I,\;\min}=\frac{\left(\zeta_{I,\;\max}-\zeta_{I,\;\min}\right)}{1+b/(K-K_{\min})}$$

with the parameters $\zeta_{I,\;\max}$ and *b* depending on the probability threshold. The relevant fitting parameters are summarized in Supplemental Table S1.

**Figure 3:**
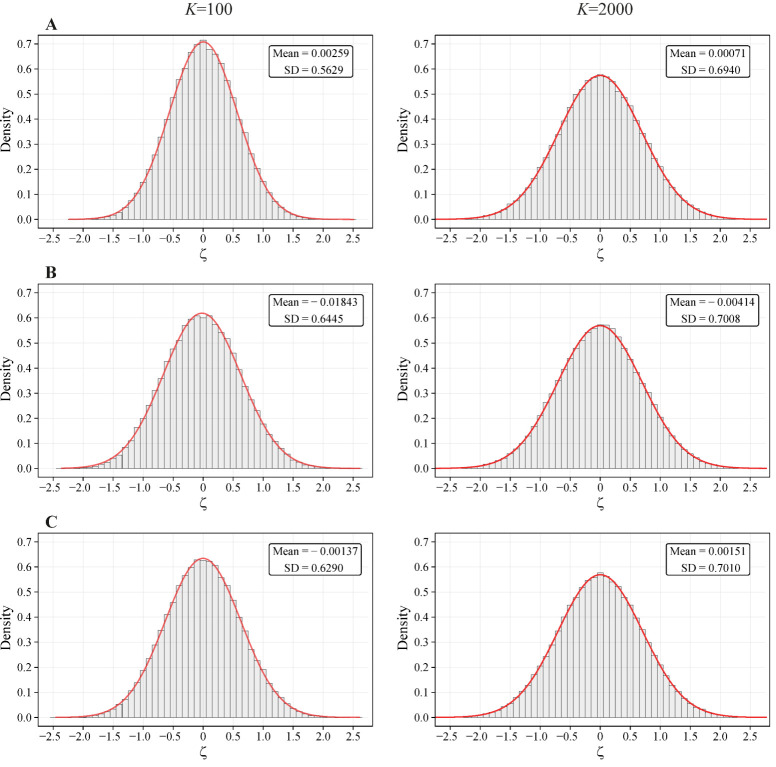
The histograms for the distributions of ζ parameters at different numbers of the nearest neighbors *K*. The histograms were obtained by 10^5^ random realizations. The best Gaussian approximations of observable distributions are shown by solid lines. The corresponding fitting parameters for Gaussian distributions are presented in the inserts. The distributions of ζ parameters for the indices of: (**A**) overlapping (Equations (1), (7) and (15)); (**B**) asymmetry (Equations (2) and (15)); (**C**) coverage (Equations (3) and (15)).

At a fixed number of nearest neighbors *K*, the distributions of $\zeta_{I}$ for all indices for the random sets appear to be close to Gaussian statistics *N*(0, σ) (see Figure 3). The mapping of the thresholds Pr = 0.05 for $\zeta_{I}$ onto Gaussian thresholds by the relationship:

(18) $$\lambda_{I}(K)\;|\zeta_{I,\;\mathrm{Pr}=0.05}(K)|\;=\;\;|z_{Gauss,\;\mathrm{Pr}=0.05}|\;=1.96$$

brings $\lambda_{I}(K)\;\zeta_{I}(K)$ parameters close to the Gaussian *z*-variables, obeying universal statistics *N*(0, 1). This similarity provides simple approximation for related *p*-values. (i) First, the actual normalized deviations $\zeta_{I}$ should be determined for the sets under analysis and compared with 5% or 1% probability thresholds for the random sets using interpolation (17). (ii) Then, mapped values $\lambda_{I}(K)\;\zeta_{I}(K)$ can be used for the approximate assessment of *p*-values by the Gaussian statistics.

### 2.4. Extension to AABB/BBAA Patterns

The previous consideration concerned the indices associated with the set of the nearest neighbors in the combinations ABA and BAB. Such a choice of neighbors was essential for the study of correlations between point–point tracks [8] and is needed for uniting previous and current analyses. We found, however, that the approach based on the indices can be extended to the pairs AB and BA in the combinations AABB and BBAA as well. All AB and BA pairs in the sets ABA–BAB and AABB–BBAA are different. Generally, the statistics of indices for the sets ABA–BAB and AABB–BBAA may also be different. Indeed, using the built-in Kolmogorov–Smirnov and Mann–Whitney criteria of the R statistics package, we revealed the statistically significant differences for some of the genome tracks studied below. Therefore, it is better to consider the sets ABA–BAB and AABB–BBAA separately. The simulations for two random tracks showed that the related 5% and 1% probability thresholds for the indices associated with the pairs AB and BA in the combinations AABB and BBAA are close to those found in Section 2.3 up to small statistical scattering. Thus, the set AABB–BBAA can be added to the general scheme and separately studied with a similar method.

The final results (overlapping/asymmetry indices and their statistical significances) depend upon the proportion between ABA–BAB and AABB–BBAA sets in the source data. For example, in most cases from the Results chapter, the ABA–BAB set contains the majority of stretches from the smaller dataset, whereas, for the TSS sets, the AABB–BBAA set contains the majority of TSS. Therefore, in our view, it is expedient in every particular case to study the colocalizations both for the ABA–BAB and AABB–BBAA sets; if the results diverge, the final decision is to be made on the basis of which of these two colocalization classes represents a larger share of the smaller source dataset.

## 3. Results

### 3.1. Test: Colocalization between Exons and Random Stretches

The statistical criteria above concern the situation when both sets of stretches are random and independent (i.e., the correlations between positions and lengths of stretches are absent). In this section, we considered the situation when only one of the stretch sets is random, while the other set is non-random. The permutations for the random set should obey the statistics described in Section 2.2 and Section 2.3, whereas the statistics related to the permutations for the non-random set may be different. We checked this statement by testing colocalization between exons and random stretches.

**Figure 4:**
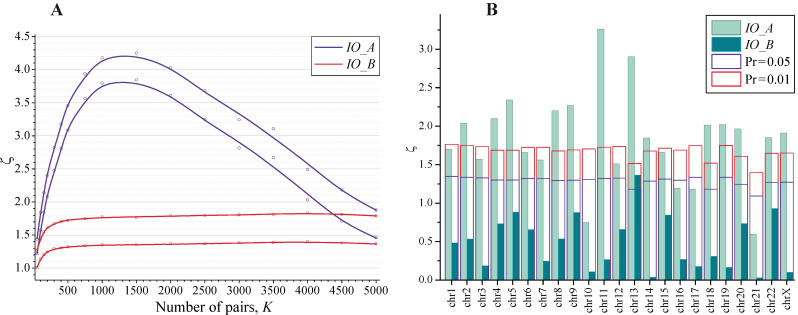
(**A**) The dependence of parameters $\left|\zeta_{IO}^{\left(a\right)}\right|$ and $\left|\zeta_{IO}^{\left(b\right)}\right|$ (Equations (1), (7), and (15)) on the number of the nearest neighbors, obtained by 10^3^ random realizations for the assessment of correlations between exons and random stretches. The upper curves correspond to Pr = 0.01 and the lower curves correspond to Pr = 0.05. (**B**) The distributions of parameters $\left|\zeta_{IO}^{\left(a\right)}\right|$ and $\left|\zeta_{IO}^{\left(b\right)}\right|$ over human chromosomes obtained for a particular random realization. The corresponding columns for the expected thresholds Pr = 0.01 (red) and 0.05 (violet) for the random sets are shown separately as a reference.

A fragment with exons was chosen on human chromosome 1, and the colocalization between exons (A-stretches) and randomly generated sets of stretches (B-stretches) were studied using indices of overlapping (6a) and (6b). As previously seen, the dependence of the statistical thresholds on the mean values $\bar{IO}$(see Equation (4)) was weak. The dependence of thresholds on the number of the nearest neighbors was studied for $\bar{IO}$ held at −0.5 and 0 approximately, with permissible variations ± 0.05. The corresponding dependences of parameters $\left|\zeta_{IO}^{\left(a\right)}\right|$ and $\left|\zeta_{IO}^{\left(b\right)}\right|$ on the number of the nearest neighbors for 5% and 1% empirical probability thresholds are shown in Figure 4A. As expected, the dependence on *K* for $\left|\zeta_{IO}^{\left(b\right)}\right|$ coincided with the random counterparts shown in Figure 2A, whereas the dependence for $\left|\zeta_{IO}^{\left(a\right)}\right|$ appeared to be strongly different. Only with a very large number of random neighbors (*K* > 5000), when the combination BAB became dominating, did the thresholds for $\left|\zeta_{IO}^{\left(a\right)}\right|$ tend towards the random criteria (see curves for *IO*_*A* in Figure 4A).

This test was also checked with the following software packages: regioneR [11], GenometriCorr [9], StereoGene [17], and Genomic Association Tester [10]. The webservice Coloc-stats [18] is a metaserver that does not offer new statistical methods for assessment of colocalization between genomic tracks and, therefore, was not tested in this work.

Test sets were generated in the following way. The set of exons on the forward strand of *H. sapiens* chromosome 1 was used as a model non-random set. An ad hoc Perl script based upon the Mersenne Twister random number generator was used to generate 1000 random datasets for stretches by the following rules: The stretch centers were distributed randomly and uniformly; the minimum and maximum coordinates of centers were concordant with those for the exons on the forward chain of the human chromosome 1; and the lengths of the stretches were distributed by random uniform distribution and varied from 1 bp to the maximum exon length on the chromosome 1. As the number of nearest neighbors between stretches of two types is most important for the statistics, the number of generated random stretches was adjusted to obtain a fixed number of nearest neighbors. All software packages shared the same datasets during testing.

These 1000 random realizations were used to determine the observable false discovery rate (FDR). For the correct assessments with *p*-values less than 0.05, FDR should also be 0.05, independent of the number of the nearest neighbors. We found, however, that the actual FDR was about twice as high for regioneR, GenometriCorr, and GAT packages and significantly depended on the reference-query selection for regioneR and GenometriCorr, whereas for StereoGene and for our Genome Track Colocalization Analyzer package, FDRs were correct (see Table 1 for a summary). As is seen from Table 1, typically, FDR exceeds the correct threshold for the packages based exclusively on overlapping stretches [9,10,11], whereas StereoGene [17], assessing the correlations between stretches/profiles, provides correct predictions. Note, however, that StereoGene calculates only correlations between genome tracks and profiles, whereas the information about the character of (non)overlapping between stretches is absent. The Genome Track Colocalization Analyzer provides this latter information as well.

We benchmarked all software packages (Table 1) for the exons on chromosome 1 and random datasets with approximately 500 colocalization pairs. All calculations were performed 100 times for each software package in single-threaded mode. Multithreaded calculations are inherent for regioneR and GAT, while other software packages can be accelerated to run in parallel for many tasks at once. We can conclude that StereoGene and the Genome Colocalization Track Analyzer are the fastest software packages and provide more correct results. We should mention, however, that StereoGene results strongly depend upon the window size parameter.

A similar test was also applied to the complete set of human chromosomes (except the Y chromosome). The indices of overlapping (6a) and (6b) were calculated for each chromosome separately for a particular random realization and compared versus 5% and 1% probability thresholds for the random sets. The numbers of the nearest neighbors *K* for the chosen random realization varied over the chromosomes from 88 to 986. The expected predictions for the random sets were calculated depending on *K* by Equation (17). The resulting histogram for the distribution of ζ parameters (taken by the absolute values) over the chromosomes is shown in Figure 4B. The probability to observe by chance *n* events where the ζ values exceed the prediction for a chosen threshold *p*-value may be assessed by binomial distribution:

(19) $$\mathrm{Pr}\;(n)=C_{n}^{N_{chr}}p^{n}{\left(1-p\right)}^{N_{chr}-n}$$

where *N_chr_* is the total number of chromosomes and $C_{n}^{N_{chr}}$ is the binomial coefficient. For *p* = 0.05 and *N_chr_* = 23, the observable number of such events should not exceed 1–3. This is actually the case for $\zeta_{IO}^{\left(b\right)}$ parameters and is drastically violated for $\zeta_{IO}^{\left(a\right)}$ parameters, as seen in Figure 4B.

To sum up, stretch–stretch colocalization should be assessed by indices defined by Equations (6a), (6b), and (7) and the respective parameters $\zeta_{IO}^{\left(a\right)}$, $\zeta_{IO}^{\left(b\right)}$, $\zeta_{IO}$ (Equation (15)). The choice between (6a), (6b), and (7) criteria should be made according to the randomness of distribution of stretches over the genome (both sets are non-randomly distributed by the statistical criterion, one set is non-randomly/one set is randomly distributed by the statistical criterion, both sets are randomly distributed by the statistical criterion). The randomness of stretch positions can be assessed by the structural entropy for the centers of stretches similarly to that developed previously for the point–point tracks [8,19]. The difference between structural entropy for the centers of stretches in datasets putatively considered random should be statistically significant in terms of the standard deviations for random entropy [8,19]. Otherwise, the positions of stretches should be considered non-random, i.e.:

- If structural entropy criterion $\left|z_{s}\right|\geq 1.96$ for each of the stretch sets (that means the centers of stretches for both sets are distributed non-randomly), then Equation (7) is applied.
- If the positions of centers for one of the stretch sets are distributed non-randomly ($\left|z_{s}\right|\geq 1.96$), whereas the other centers are distributed randomly ($\left|z_{s}\right|<1.96$), the general colocalization should be assessed via either criterion (6a) or (6b) (and the respective $\zeta_{IO}$) for the random set.
- If the positions of centers for both stretch sets are random ($\left|z_{s}\right|<1.96$ for each set), then Equation (7) is applied again.

If the absolute value of the $\left|\zeta_{IO}\right|$ parameter, as calculated by the selected equation, appeared to be higher than the respective threshold corresponding to Pr = 0.05 for the random sets, the stretch–stretch colocalizations were assumed to be significant; otherwise, colocalizations were assumed to be insignificant. The comprehensive approach consists of the combined set of Equations (6a), (6b), and (7) and the additional estimation of center positioning randomness for subsequent expert assessment as described above.

We implemented the Genome Track Colocalization Analyzer in Perl with the XS extension module to speed up calculations. The current version is a command line utility that depends upon the following CPAN modules (Sort::Key, Math::Random::MT::Auto, Config::Tiny, PerlIO::gzip, Getopt::Long, Math::Round, Math::CDF, Math::Interpolate) and provides output both in the text and HTML formats, facilitating the integration of output data into both bioinformatic pipelines and web servers.

### 3.2. Colocalization between Stretches and Gene Expression

We applied the software we developed to the study of the colocalization and length asymmetry of particular genome tracks related to the regulation of gene expression. All colocalization studies in this work were carried out with the *H. sapiens* genome build GRCh38/hg38 p.12. The relevant genome tracks data processing methods and accession numbers can be found in Supplemental Methods.

The first three examples that we present in this work include CpG islands. Their colocalization with exons [20], transcription start sites (TSS) [20,21,22,23], and DNAseI Hypersensitive sites (DHSS) [24,25] was previously affirmed experimentally. We observed statistically significant colocalization by our method in all these cases as well. The concluding example concerns the colocalization of H2A.Z (H2AFZ) histone mark and transcription start sites (TSS) in the *H. sapiens* genome. As a result, we revealed the statistically significant colocalization of the H2A.Z histone mark with the bidirectional promoters in K562 cell line. The colocalization of the H2A.Z histone mark with the bidirectional promoters was compared with that for unidirectional and silent TSS in the same cell line.

#### 3.2.1. Strong Colocalization between CpG Islands and Exons Suggests a Role of CGI in Transcription

**Figure 5:**
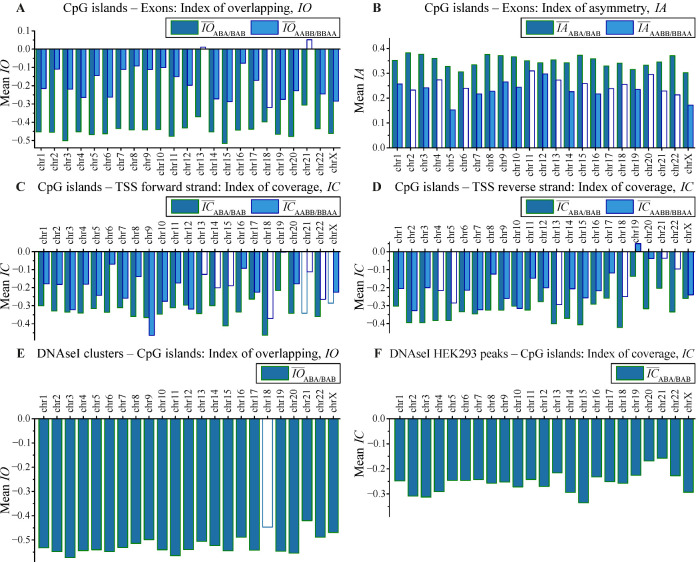
Distribution over human chromosomes of the mean indices for the different stretches. Mean index of: (**A**) overlapping (Equation (1)) for CpG islands and exons; (**B**) asymmetry (Equation (2)) for CpG islands and exons; (**C**) coverage (Equation (3)) for CpG islands and TSS on the forward strand; (**D**) coverage (Equation (3)) for CpG islands and TSS on the reverse strand; (**E**) overlapping (Equation (1)) for DNAseI clusters and CpG islands; (**F**) coverage (Equation (3)) for DNAseI HEK293 peaks and CpG islands. Non-filled bars indicate chromosomes for which the index values are not statistically significant (|*z*| < 1.96, *p* > 0.05).

**Figure 6:**
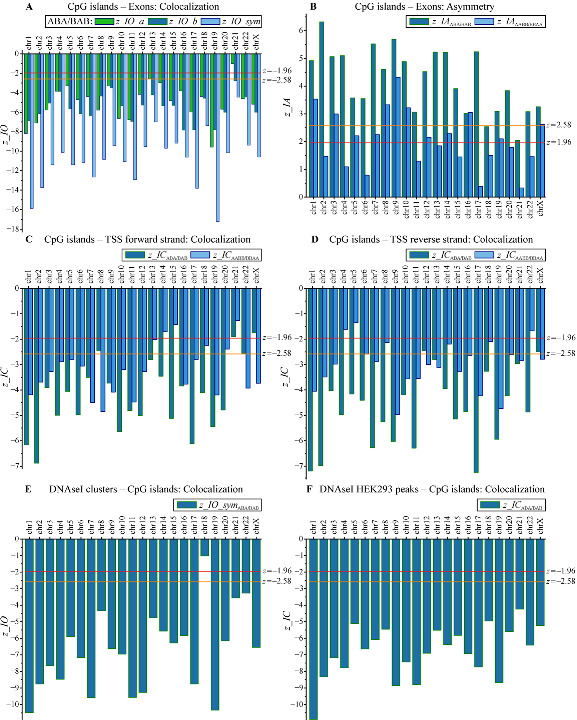
Distribution over human chromosomes of the Gaussian *z*-variables for the assessment of statistical significance of the mean indices on Figure 5 by permutation tests. The thresholds for probabilities Pr = 0.05 and 0.01 are shown by horizontal lines (*z* = ±1.96 and *z* = ±2.58, respectively). The Gaussian *z*-variables for the assessment of statistical significance of mean indices for: (**A**) CpG islands and exons, by Equations (6) and (7); (**B**) CpG islands and exons, by Equation (8); (**C**) CpG islands and TSS on the forward strand, by Equation (9); (**D**) CpG islands and TSS on the reverse strand, by Equation (9); (**E**) DNAseI clusters and CpG islands, by Equation (7); (**F**) DNAseI HEK293 peaks and CpG islands, by Equation (9).

The colocalizations between CpG islands (CGIs) and exons were previously studied experimentally [20,26]. The software we developed was applied to the analysis of colocalization between CGIs and *H. sapiens* exon genome tracks. The results presented in Figure 5A and Figure 6A demonstrate the statistically significant colocalization between CGIs and exons according to all criteria (6a), (6b), and (7) for the vast majority of the chromosomes. The *p*-values of < 0.01 for each test resulted in much lower integral estimation calculated by Equation (19). The mean values of the colocalization index $\bar{IO}$ were negative for all chromosomes. The results of length asymmetry analysis presented in Figure 5B and Figure 6B demonstrate that for almost all chromosomes, there was significant length asymmetry. For the correlation pairs ABA/BAB, this asymmetry was significant for all chromosomes, except chrY, whereas for the correlation pairs AABB/BBAA, the asymmetry was significant for 12 chromosomes. For three chromosomes, the number of correlation pairs was too small for statistics. CGI stretches were longer than exon stretches ($\bar{IA}$ > 0.3, the significance of obtained indices corresponded to *p* < 0.01 for the vast majority of chromosomes).

Colocalization patterns for ABA/BAB and AABB/BBAA correlation pairs matched completely for the vast majority of chromosomes (excluding chr13). The statistical significance of colocalization for the vast majority of chromosomes was at the level *p* < 0.01.

This result was confirmed by existing experimental data [20], suggesting that CGIs play a role in regulation of transcription. Additional information can be found in Supplemental Table S2.

#### 3.2.2. Strong Colocalization between CpG Islands and Transcription Start Sites Confirms CGIs Take Part in Transcription Regulation

It is known that CGIs are often associated with mammalian promoters and may even be used as alternative promoters for the genes with methylated promoters [27,28]. The colocalization of CGIs with TSS affects gene expression in eukaryotes [28]. The results for the colocalization analysis obtained with our package are shown in Figure 5C,D and Figure 6C,D and confirm statistically significant colocalization between CGIs and TSS. The related colocalization indices $\bar{IC}$ are negative for almost all chromosomes simultaneously for forward and reverse strands for both ABA/BAB and AABB/BBAA datasets; this means that CpG stretches and TSS points are frequently intersected ($\bar{IC}$≈ −0.3 genome-wide). The statistical significance of $\bar{IC}$ indices corresponds to *p* < 0.01 for the vast majority of chromosomes for both forward and reverse strands. The difference between the results for ABA/BAB and AABB/BBAA datasets was observed only in the cases when the number of correlation pairs was too small for statistics (in particular, the amount of correlation pairs < 50 was observed for 13, 18, 21, and 22 chromosomes).

The obtained results are supported by existing experimental data [20,21,22,23] and independently confirm the conclusion that CGIs are involved in the regulation of human cells transcription. The additional detailed information for the genome-wide analysis can be found in Supplemental Table S3.

#### 3.2.3. Strong Colocalization between CpG Islands and DNAseI Hypersensitivity Sites Suggests That CGIs Often Correspond to Open Chromatin Regions

The colocalization between CGIs and DNAseI hypersensitive sites (DHSS) was studied earlier and revealed that some DHSS are associated with CGIs [24]. It is also known that DHSS are often located near TSS [25]. This is why we chose to search for colocalizations between CGI and DHSS using our software. We used two genomic tracks for DHSS: clusters of DNaseI hypersensitivity sites derived from assays in 95 cell types (wgEncodeRegDnaseClustered) and HEK293 DHSS peaks (ENCFF127KSH). Statistical trials were performed in the stretch–stretch mode for DHSS clusters and in the stretch–point mode for HEK293 DHSS peaks (due to the fixed size of HEK293 DHSS peaks).

In both cases, we detected statistically significant colocalizations between DHSS and CGIs. For DHSS clusters ($\bar{IO}$≈ −0.5 genome–wide), the significance of colocalization was at the level *p* <0.01 for the vast majority of chromosomes. Similar results were obtained for HEK293 DHSS peaks ($\bar{IC}$≈ −0.25 genome-wide, i.e., the centers of HEK293 DHSS peaks are located within CGI stretches). *H. sapiens* genome hg38 contains 27,949 CGIs that form 26,741 correlation pairs with DHSS clusters, of which 24,589 pairs have direct overlaps. This means that 87.9% of CGIs overlap significantly with DHSS clusters derived from assays in 95 cell types. We can conclude that the vast majority of CGIs are associated with DHSS. These data strongly suggest that CGIs in human cells almost always correspond to open chromatin regions.

For AABB/BBAA set pairs, the number appears to be lower than the acceptable statistics threshold (<50) for almost all chromosomes for DHSS clusters–CGIs colocalizations. For the HEK293 DHSS peaks–CGIs case, the data amount in ABA/BAB correlation pairs exceeded the data amount in AABB/BBAA correlation pairs by almost a factor of 9 (68.8% in ABA/BAB, 7.9% in AABB/BBAA), which makes AABB/BBAA colocalization results negligible. This case is a good example showing that statistics for ABA/BAB and AABB/BBAA correlation pairs can be quite different, and therefore these datasets should be analyzed separately.

The detailed data can be found in Figure 6E,F and in Supplemental Table S4.

#### 3.2.4. Genome-Wide Study of Colocalization between Promoters and Histone Mark H2A.Z (Isoform H2AFZ) for Cell Line K562

The genomes of eukaryotes contain a fairly large number of gene pairs for which the transcription is moving to non-intersecting directions on the different DNA strands. If a distance between TSSs of corresponding promoters is less than 1000 bp, a DNA fragment between related TSSs is called a “bidirectional promoter”. For brevity, we designated TSS associated with bidirectional promoter as bTSS; otherwise, TSS is designated as uTSS. In our previous work [8], we found significant correlations between TSSs on the direct and reverse strands in the genomes of *D. melanogaster*, *C. elegans*, *M. musculus*, *H. sapiens*, and *D. rerio* (*p* < 0.01 for the vast majority of chromosomes). We also found statistically significant positional correlations between them, with TSSs on the reverse strand preceding TSSs on the direct strand. As the factors regulating gene expression are usually located before TSS (on each of the strands), such coordinate and positional correlations between TSSs indicate that regulatory elements such as CpG-islands, enhancers, silencers, etc., can be common and shared for these TSSs. Statistical significance of the detected TSSs correlations supports the positive selection of such expression mode during molecular evolution. Indeed, such a mode of expression regulation was confirmed experimentally [29,30,31]. Despite the progress in the study of bidirectional promoters, the details of the relevant molecular features remain in part controversial and require further investigation.

In the work [32], we noticed that the DNA double-strand breaks (DSBs) profile around TSSs for the EPD database (which contains only experimentally validated promoters and TSSs) is quite similar to transcription factor occupancy profile around TSSs [33]. Replacement of canonical histones by histone variants, in particular, the replacement of H2A by H2A.Z (isoform H2AFZ) over promoters [34,35,36], plays an important role in chromatin dynamics during transcription and other DNA-templated activities. It is also known that the histone variant H2A.Z frequently accumulates around TSS and enhancer elements, indicating its involvement in transcription regulation [35,37,38,39,40,41,42,43,44]. In addition, the histone variant H2A.Z localizes preferentially in heterochromatin and nearby DSB sites [44,45,46,47,48,49]. Biochemical studies revealed that H2A.Z form nucleosomes with multiple positions [50,51]. In this section, we study the colocalization of H2A.Z signal peaks and TSSs.

The analysis was performed for TSSs taken from the following databases: EPD_new [3], Gencode [4], RefSeq [52], and EMBL for the *H. sapiens* cell line K562. For each database, we first divided TSSs for silent and active genes, and then additionally subdivided TSSs corresponding to bidirectional and unidirectional promoters as defined above. The extended peaks for H2AFZ K562 were taken from EncodeProject accession ENCSR000APC replicated peaks ENCFF921IKK. The colocalization was assessed with indices of coverage, with TSSs being treated as points and extended peaks for H2AFZ being treated as stretches. The detailed description of this procedure can be found in Supplemental Methods.

At the first step, using the Genome Track Analyzer package [8], we studied the correlations between TSSs and the centers of signal peaks for histone modification H2A.Z (isoform H2AFZ), replacing histone H2A in the nucleosome. We found a significant correlation (*p* < 10^–6^) for all TSSs related to active genes. Similar results were obtained using StereoGene [17] (Supplemental Table S5).

**Figure 7:**
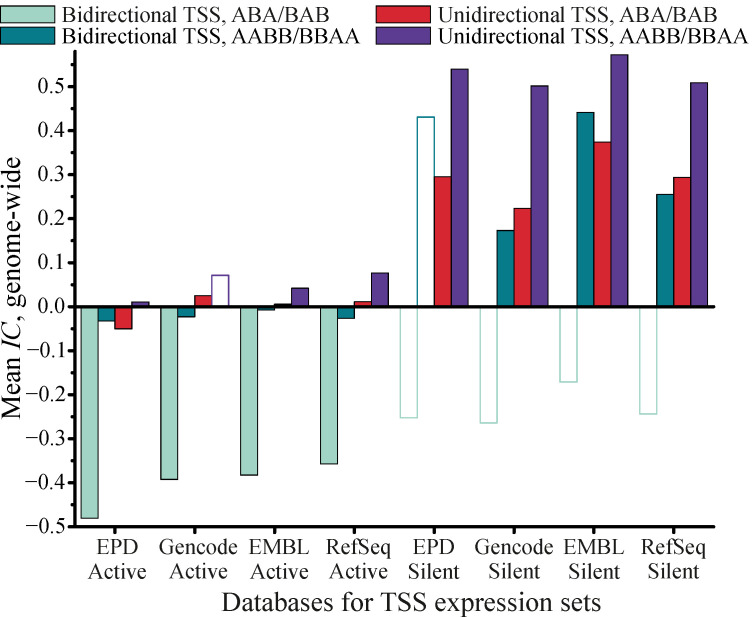
Genome-wide comparison of indices of coverage for colocalization between histone isoform H2AFZ and bi- and unidirectional TSS related to active and silent genes for cell line K562. Non-filled bars indicate the indices that are not statistically significant (|*z*| < 1.96, *p* > 0.05).

In the second step, we studied colocalization between bTSSs/uTSSs and extended the signal peaks for H2A.Z using the Genome Track Colocalization Analyzer package (Supplemental Table S6). We found a statistically significant colocalization with overlapping ($\bar{IC}$< −0.35, *p* < 10^–6^) between bTSSs and H2A.Z peaks for active genes. We found a statistically significant trend for active genes uTSSs to be located near H2A.Z peaks, typically without overlapping these peaks ($\bar{IC}$ > 0.01, i.e., the overlapping index is positive but close to 0). For uTSSs related to silent genes, we found a statistically significant trend for colocalization with H2A.Z peaks without overlapping ($\bar{IC}$ > 0.2, *p* < 10^–6^). Figure 7 illustrates the clear difference in the trends for colocalizations between bTSSs/uTSSs and H2A.Z peaks for active and silent genes. The trends persisted throughout all four TSS databases and ABA/BAB or BBAA/AABB grouping.

The results we obtained demonstrate that in order to reach reliable conclusions about mutual colocalization of genome tracks, it is not sufficient to apply correlation-based GWAS tools. It is necessary, however, to create and implement specific methods for assessing the statistical significance of genomic track colocalization and overlapping (overlapping indices).

For example, it is impossible to make a conclusion about colocalization of the H2A.Z genome track and various TSSs subsets by applying correlation-only GWAS tools (Supplemental Table S5), whereas our new method provides comprehensive results.

Our results indicate that colocalization between TSSs and H2A.Z may facilitate the transcription initiation via freeing promoters from nucleosomes. The replacement of histone H2A by H2A.Z makes the nucleosome less stable [53] and allows RNA-polymerase II to shift or decompose the nucleosome.

Bidirectional promoters have a statistically significant trend to overlap with H2A.Z peaks; however, nucleosome colocalization with one of the TSS of the bidirectional promoter does not obstruct transcription start from the other TSS. We suggest that no nucleosomes containing H2A.Z are to be found near the TSSs of silent genes and, therefore, RNA-polymerase II is unable to shift the nucleosome and to start the transcription. Note that for bidirectional promoters, the distance between TSS and the middle of the H2A.Z peak tends to be approximately 100–200 bp (Supplemental Figure S1), matching the promoter NDR (nucleosome-depleted region) width. These conclusions agree with the observations by [54], who proved the relationship between positioning of H2A.Z (H2AFZ) nearby TSS with the expression level. We plan to continue this research in future works by studying the “wide promoters” class and H2A.Z peak colocalizations, as well as by varying the expression threshold for active/silent genes.

## 4. Discussion

The method developed in our paper for the study of colocalization between stretch-like genome tracks, as well as the Genome Track Colocalization Analyzer (GTCA) package based on this method, provide an efficient tool for bioinformatic analysis of various genetic mechanisms related to the gene expression. Its reliability and calculation speed proved to be on the level of the top available packages (see Table 1). The mapping of stretch parameters onto the indices varying within the interval (−1, +1) ensures the robustness of the results to inhomogeneity of track distribution over the genome and to variations in positioning and lengths of stretches. The user does not need to tune the parameters related to the selection of representatives in a sampling set and the number of trials, as is typical for partial permutation tests.

In fact, any stretch set subdivides the genome into two sets, original and complementary (or void) ones. In some cases, the mutual absence of features (or colocalization between void sets) may also be of interest. The colocalization between presence/absence of some features can be considered as an equivalent of correlations/anticorrelations.

Certain other problems can also be efficiently reduced through the study of stretches. In particular, coarse-graining of various profiles (e.g., AT/GC content, expression profiles, protein binding profiles) results in a set of stretches within which the characteristics exceed some thresholds. Previously, it was shown that the coarse-grained profiles obtained by transitional automorphic mapping of the genome on itself (TAMGI) contain important information about functional regions in viral genomes [55,56]. The colocalization between stretch sets obtained by coarse-graining of profiles can also be treated by the method developed in our paper. This extends the scope of applications of the methods that we describe.

## 5. Conclusions

Combining the method and package developed for the study of colocalization between stretch-like genome tracks with those previously developed for the study of the correlation among point-like tracks [8] provides a complete analysis of relationships between genomic tracks. The united method can be applied to the general scope of genome-wide association studies (GWAS) and/or can be used as a particular option in bioinformatic pipelines. The relevant bioinformatic analysis can be used for data mining and may stimulate further experimental studies.

## Figures and Tables

**Table 1:** FDR for the tests on the absence of correlations between exons on the forward strand of human chromosome 1 and random sets.

	Fraction of Events with Predicted *p* < 0.05 per 1000 MC Realizations			Benchmark, Time per 1 Run
Option	Pairs ≈ 50	Pairs ≈ 500	Pairs ≈ 5000	Pairs ≈ 500
regioneR [11]				
overlapPermTest *p*-value, exons–set *A*; random–set *B*	0.024	0.082	0.093	543.6 s
region distance *p*-value, exons–set *A*; random–set *B*	0.871	0.772	1.000	
overlapPermTest *p*-value, random–set *A*; exons–set *B*	0.026	0.080	0.051	543.6 s
region distance *p*-value, random–set *A*; exons–set *B*	0.092	0.100	0.056	
GenometriCorr [9]				
projection test *p*-value, exons–reference; random–query	0.081	0.120	0.072	72.94 s
Jaccard test *p*-value, exons– reference; random–query	0.065	0.105	0.074	
projection test *p*-value, random–reference; exons–query	0.090	0.088	0.098	72.94 s
Jaccard test *p*-value, random–reference; exons–query	0.066	0.106	0.091	
Genomic Association Tester [10]				
gat-run.py *p*-value, exons–annotation; random–segment	0.067	0.108	0.080	24.96 s
gat-run.py *p*-value, random–annotation; exons–segment	0.068	0.109	0.093	106.56 s
StereoGene [17]				
Mann *Z*-criterion, exons–reference; random–query, wSize = 5000	0.046	0.050	0.050	0.235 s
Mann *Z*-criterion, random–reference; exons–query, wSize = 5000	0.046	0.047	0.053	0.235 s
Genome Colocalization Track Analyzer				
United ζ-criterion, Equations (6), (7) and (15)	0.049	0.052	0.050	0.233 s

Abbreviations: FDR, false discovery rate; MC, Monte Carlo. All tests were performed with predicted *p*-values less than 0.05. The expected mean value and standard deviation for FDR per 1000 MC realizations should be 0.05 ± 0.007.

## Data Availability

The applications for Linux, MacOS X, and Windows are available as https://www.mdpi.com/article/XXXXXXXX/s2 and at http://ancorr.eimb.ru (accessed on 28 August 2022). The source codes are available at Github: https://github.com/lokapal/GTCA2022 (accessed on 28 August 2022).

## Supplementary Materials

The supporting information can be downloaded at: https://www.mdpi.com/article/10.3390/biology11101422/s1. Figure S1: Comparison of H2AFZ signals for bi- and unidirectional promoters, both expressing and silent, around K562 TSS; Table S1: The dependence of statistical thresholds for ζ parameters on the number of pairs and on the mean indices; Table S2: Chromosome- and genome-wide colocalizations between CpG Islands and exons for *H. sapiens* GRCh38p13/hg38 genome build; Table S3: Chromosome- and genome-wide colocalizations between CGIs and TSSs for *H. sapiens* GRCh38p13/hg38 genome build; Table S4: Chromosome- and genome-wide colocalizations between CpG Islands and DNAseI clusters/peaks for *H. sapiens* GRCh38p13/hg38 genome build; Table S5: Genome-wide correlations by the Genome Track Analyzer/StereoGene between H2AFZ and TSSs subsets for K562 *H. sapiens* cell line; Table S6: Genome-wide colocalizations between various TSS and H2AFZ for K562 *H. sapiens* cell line; Supplemental Methods.
