# Supplementary material for: Genome-Wide Study of Colocalization between Genomic Stretches: A Method and Applications to the Regulation of Gene Expression
Source: Biology (Basel). 2022 Sep 29;11(10):1422. doi: 10.3390/biology11101422 (PMC9598420; doi:10.3390/biology11101422)
Supplement: Supplementary file 1 [file biology-11-01422-s001.zip › Supplemental_Figure_S1.pdf]

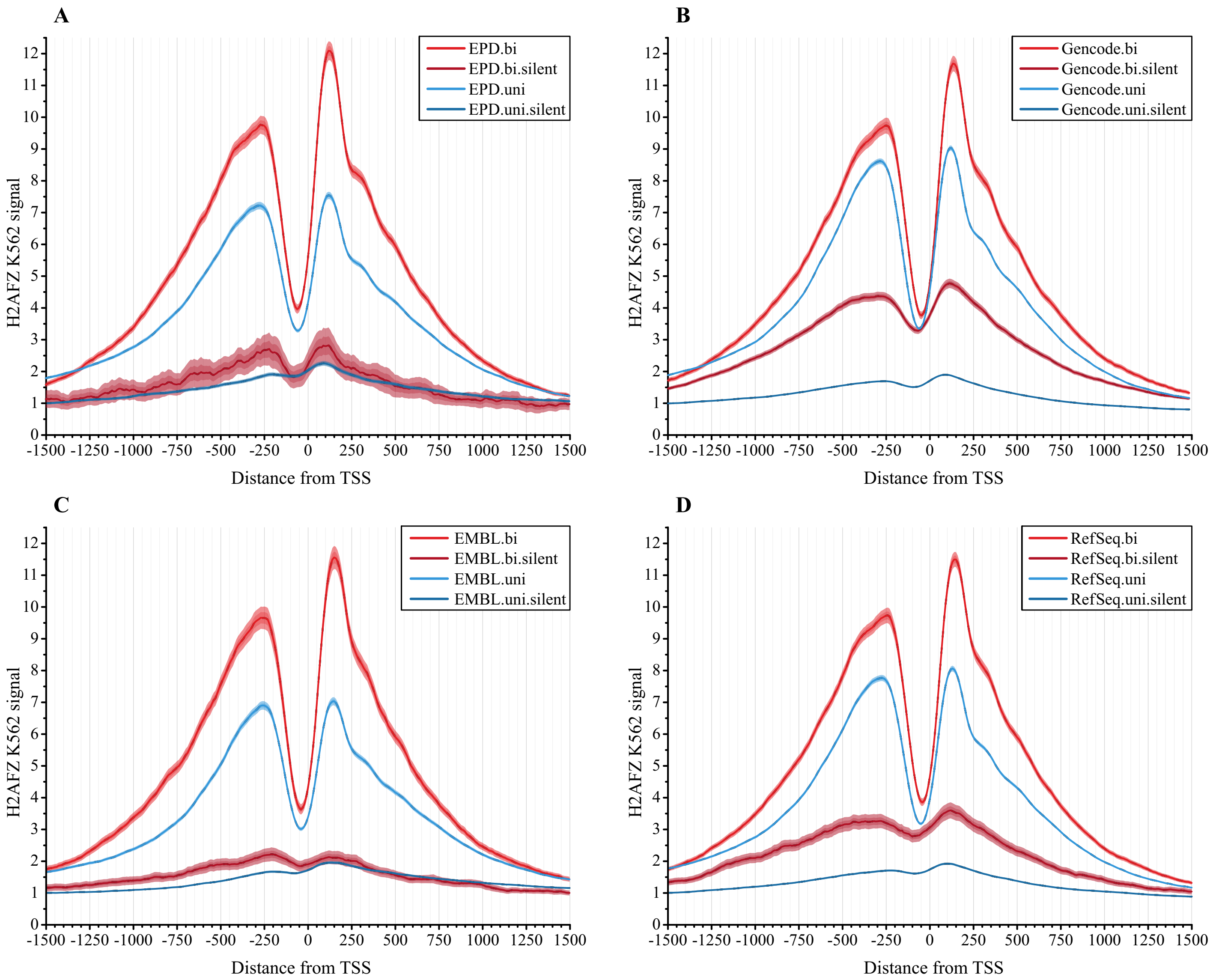

Supplementary Figure S1. Comparison of H2AFZ signals for bi- and unidirectional promoters, both expressing and silent, around K562 TSS. **A**, H2AFZ signals for EPD TSS database. **B**, H2AFZ signals for Gencode TSS database. **C**, H2AFZ signals for EMBL TSS database. **D**, H2AFZ signals for RefSeq TSS database.
