# Supplementary material for: Genome-Wide Study of Colocalization between Genomic Stretches: A Method and Applications to the Regulation of Gene Expression
Source: Biology (Basel). 2022 Sep 29;11(10):1422. doi: 10.3390/biology11101422 (PMC9598420; doi:10.3390/biology11101422)
Supplement: Supplementary file 1 [file biology-11-01422-s001.zip › Supplemental_Methods.pdf]

Supplemental Methods. Preparation and processing of datasets

All calculations in the paper were performed with *H.sapiens* GRCh38/hg38 p.12 genome layout. *H. sapiens* hg38 exons genome track was obtained by the following way:
Exons: Raw exons data was downloaded from the NCBI RefSeq Curated database [1] at UCSC Genome Browser server by the command:
`mysql --user=genome -N --host=genome-mysql.cse.ucsc.edu -A -D hg38 -e "select chrom, txStart, txEnd, strand, exonCount, exonStarts, exonEnds, name2 from ncbiRefSeqCurated order by chrom,txStart"`
Then all exons that don't belong to the mapped chromosomes (located in alternate contigs, unlocalized sequences, unplaced sequences) were removed. At the next step we removed the complete copies of existing exons (names and strand are matched, coordinates are the same, or one of the exons is located inside the other exon completely). If the coordinates matches (or one of exons is located inside the other exon completely) while names are different then only one exon is left in the list with both names, separated by ":" sign. At the last step some exons from the same genes that overlapped were merged by the following rule: if exons belong to the same gene and if one of exon's start or end is located closer than 11 nt (1 DNA helix coil) to the other exon's start or end then these two exons are merged. At the last processing step we have added Ensemble gene IDs to the exons list by the R script. The complete hg38 exons genome track and all scripts applied to obtain it are located at Github: https://github.com/lokapal/hg38exons

Transcription Start Sites (TSS). Raw TSSs datasets were downloaded from 4 databases: EPD_new [2]: ftp://ccg.epfl.ch/epdnew/H_sapiens/current/Hs_EPDnew.sga; Gencode [3]: http://www.ensembl.org/biomart/martview (Ensembl Genes, Dataset Human genes, Filters Attributes: Chromosome/scaffold name, Transcription start site (TSS), Strand, Gene stable ID); RefSeq [1]: `mysql --user=genome -N --host=genome-mysql.cse.ucsc.edu -A -D hg38 -e "select chrom, txStart, txEnd, strand, name2 from wgEncodeGencodeBasicV38 order by chrom,txStart"`; EMBL: ftp://ftp.ensembl.org/pub/release-104/gtf/homo_sapiens/Homo_sapiens.GRCh38.104.chr.gtf.gz . All TSSs that don't belong to the mapped chromosomes (located in alternate contigs, unlocalized sequences, unplaced sequences) were removed.
Then raw data were processed uniformly: at the first step we have removed complete duplicates and joined TSSs with the same coordinate that belong to different genes. All silent in K562 cells TSSs were removed from the datasets for cell-specific epigenetics colocalization calculations in the following way: CAGE expression data for K562 cells hg38 genome build were downloaded from EPD [2] FTP server:

ftp://ccg.epfl.ch/mga/hg38/fantom5/chronicMyelogenousLeukemiaCellLine_aK562ENCODE_cBiol_rep1.CNhs12334.10824-111C5.hg38.nobarcode.sga.gz
ftp://ccg.epfl.ch/mga/hg38/fantom5/chronicMyelogenousLeukemiaCellLine_aK562ENCODE_cBiol_rep2.CNhs12335.10825-111C6.hg38.nobarcode.sga.gz
ftp://ccg.epfl.ch/mga/hg38/fantom5/chronicMyelogenousLeukemiaCellLine_aK562ENCODE_cBiol_rep3.CNhs12336.10826-111C7.hg38.nobarcode.sga.gz
and averaged. The TSS assigned to be silent if TSS has no defined expression value and neighboring ± 5 nt coordinates have no expression values too. Then we applied the following filter to the GENCODE, RefSeq and EMBL TSS data: the major TSS for each gene was identified and other TSSs that have expression values below 5% of the major TSS were removed. At last each TSS list was divided to the bidirectional TSSs (i.e. TSS that are located at the range less 1000 nt and are divergent) and unidirectional (all other TSSs). From the unidirectional TSSs list were removed TSSs from the genes that were identified as bidirectional. Genome track was created with stretches consisting of bidirectional TSSs pairs additionally. The processed hg38 TSS genome tracks for K562 and all scripts applied to obtain them are located at GitHub: https://github.com/lokapal/hg38TSS.K562

CpG: CpG islands hg38 genome track was downloaded from UCSC Genome browser HGtables server, accession cpgIslandExt: `mysql --user=genome -N --host=genome-mysql.cse.ucsc.edu -A -D hg38 -e "select chrom, chromStart, chromEnd from cpgIslandExt order by chrom,chromStart"`

DNAseI clusters. Clusters of DNaseI hypersensitivity derived from assays in 95 cell types as part of the ENCODE project [4] first production phase were downloaded from UCSC Genome browser hgTables server, accession wgEncodeRegDnaseClustered:

```
mysql --user=genome -N --host=genome-mysql.cse.ucsc.edu -A -D hg38 -e "select chrom, chromStart, chromEnd, score from wgEncodeRegDnaseClustered order by chrom,chromStart"
```

DNAseI for HEK293T. EncodeProject accession ENCSR000EJR, peaks ENCFF127KSH, hotspots ENCFF552YLW.

H2AFZ for K562: EncodeProject accession ENCSR000APC replicated peaks ENCFF921IKK, fold change over control profile ENCFF494WCA.

Genome-wide correlation assessments (Supplemental Table S5): Genome Track Analyzer v.1.3 [5] was applied with default options, StereoGene v.2.20 [6] was applied with window size 10000 (option –w 10000).

Genome-wide colocalization assessments: All dataset were processed uniformly by Genome Track Colocalization Analyzer command-line utility with the following options:

```
--gw                genome-wide correlations processing type
--pairtype      -pt correlation pairs type: 0-ABA/BAB, 1-AABB/BBAA, 2-both
--pairlimit     -pl correlation pairs above pairlimitsize will be omitted
--pairlimitsize –ps set maximum correlation pair limit length (default 10000)
```

E.g. for H2AFZ and K562 Gencode bidirectional TSS assessment the commands were the following:
ABA/BAB colocalization assessment:

```
GTCA.pl -gw -i1 H2AFZ.K562.bed -f1 0 -i2 TSS_hg38_gencode.bidi.sgr -f2 2
-o H2AFZ-TSS.ABA.gencode.bidi.txt -pl -ps 10000 -pt 0
```

AABB/BBAA colocalization assessment:

```
GTCA.pl -gw -i1 H2AFZ.K562.bed -f1 0 -i2 TSS_hg38_gencode.bidi.sgr -f2 2
-o H2AFZ-TSS.AABB.gencode.bidi.txt -pl -ps 10000 -pt 1
```

References

1. O'Leary, N.A.; Wright, M.W.; Brister, J.R.; Ciufo, S.; Haddad, D.; McVeigh, R.; Rajput, B.; Robbertse, B.; Smith-White, B.; Ako-Adjei, D., *et al.* Reference sequence (RefSeq) database at NCBI: current status, taxonomic expansion, and functional annotation. *Nucleic Acids Res* **2016**, *44*, D733-745.
2. Dreos, R.; Ambrosini, G.; Groux, R.; Cavin Perier, R.; Bucher, P. The eukaryotic promoter database in its 30th year: focus on non-vertebrate organisms. *Nucleic Acids Res* **2017**, *45*, D51-D55.
3. Frankish, A.; Diekhans, M.; Ferreira, A.M.; Johnson, R.; Jungreis, I.; Loveland, J.; Mudge, J.M.; Sisu, C.; Wright, J.; Armstrong, J., *et al.* GENCODE reference annotation for the human and mouse genomes. *Nucleic Acids Res* **2019**, *47*, D766-D773.
4. Davis, C.A.; Hitz, B.C.; Sloan, C.A.; Chan, E.T.; Davidson, J.M.; Gabdank, I.; Hilton, J.A.; Jain, K.; Baymuradov, U.K.; Narayanan, A.K., *et al.* The encyclopedia of DNA elements (ENCODE): data portal update. *Nucleic Acids Res* **2018**, *46*, D794-D801.
5. Kravatsky, Y.V.; Chechetkin, V.R.; Tchurikov, N.A.; Kravatskaya, G.I. Genome-wide study of correlations between genomic features and their relationship with the regulation of gene expression. *DNA Res* **2015**, *22*, 109-119.
6. Stavrovskaya, E.D.; Niranjan, T.; Fertig, E.J.; Wheelan, S.J.; Favorov, A.V.; Mironov, A.A. StereoGene: Rapid estimation of genome-wide correlation of continuous or interval feature data. *Bioinformatics* **2017**, *33*, 3158-3165.
