# Supplementary material for: Genome-Wide Study of Colocalization between Genomic Stretches: A Method and Applications to the Regulation of Gene Expression
Source: Biology (Basel). 2022 Sep 29;11(10):1422. doi: 10.3390/biology11101422 (PMC9598420; doi:10.3390/biology11101422)
Supplement: Supplementary file 1 [file biology-11-01422-s001.zip › Supplemental_Table_S1.pdf]

Supplementary Table S1. The dependence of statistical thresholds for ζ-parameters on the number of pairs and on the mean indices

A. Fitting ζ-parameters for the dependence of statistical thresholds on the number of pairs for the nearest neighbors

	b	*b*, SE	ζ_{min}	ζ_{max}	ζ_{max}, SE	Reduced chi-sq	Residual sum of sq
Pr=0.05							
IO	79.7832	2.51977	0.9049	1.3790	0.0022	3.5744E-05	5.3615E-04
IA	62.9988	2.46109	1.1167	1.3829	0.0014	1.5524E-05	2.3285E-04
IC	65.2577	2.26654	1.0936	1.3828	0.0014	1.4729E-05	2.2094E-04
Pr=0.01							
IO	94.3873	3.90313	1.1951	1.81807	0.00402	1.1229E-04	1.6800E-03
IA	90.6727	5.55811	1.4677	1.82531	0.00337	8.0247E-05	1.2000E-03
IC	68.9490	2.50666	1.4090	1.81639	0.00206	3.2872E-05	4.9308E-04

The fitting parameters were assessed by Eq. (17) for 10^5 random realizations at each *K*. Mean indices were about zero; K_{min} and ζ_{min} were fixed during approximation procedure and K_{min} was 50; SE is the standard error.

B. Fitting parameters for the dependence of statistical thresholds on the mean indices

	a	*a*, SE	Reduced chi-sq	Residual sum of sq
Pr=0.05				
IO	1.3346	0.00497	4.1931E-04	6.7100E-03
IA	1.3326	0.00246	1.0311E-04	1.6500E-03
IC	1.3591	0.00200	6.4101E-05	9.6151E-04
Pr=0.01				
IO	1.7494	0.00620	6.4948E-04	1.0390E-02
IA	1.7484	0.00330	1.8583E-04	2.9700E-03
IC	1.7810	0.00340	1.8502E-04	2.7800E-03

The dependence on the mean indices (Eq. (4)) of thresholds for the absolute values of ζ-parameters (Eq. (15)) was taken as $|\zeta| = a$ (constant). The fitting parameters were assessed for 10^5 random realizations at each mean value of indices *I*. The number of pairs for the nearest neighbors was fixed and equal to 580.
